# Contribution of Basophils to Cutaneous Immune Reactions and Th2-Mediated Allergic Responses

**DOI:** 10.3389/fimmu.2015.00393

**Published:** 2015-08-03

**Authors:** Atsushi Otsuka, Kenji Kabashima

**Affiliations:** ^1^Department of Dermatology, Kyoto University Graduate School of Medicine, Kyoto, Japan; ^2^PRESTO, Japan Science and Technology Agency, Kawaguchi, Saitama, Japan

**Keywords:** basophil, dendritic cell, Th2, contact dermatitis, IgE-CAI

## Abstract

Basophils are potent effector cells of innate immunity and also play a role in T helper 2 (Th2)-mediated allergic responses. But, although their *in vitro* functions are well studied, their *in vivo* functions remain largely unknown. However, several mouse models of basophil depletion have recently been developed and used to investigate basophil functions. For example, in a croton oil-induced model of irritant contact dermatitis in conditionally basophil-depleted transgenic mice, we found that basophils rapidly infiltrate inflamed skin and subsequently induce infiltration of eosinophils. We also showed that basophils induce Th2 skewing upon epicutaneous sensitization with various haptens and peptide antigens. Intriguingly, basophils also promoted Th2 polarization upon protein antigen exposure in the presence of dendritic cells (DCs). The dermal DC subset associated with Th2 skewing was recently identified as CD301b^+^ DC. Such studies with basophil-deficient mouse models have significantly improved our understanding of the mechanisms involved in human immune-related diseases. In this review, we will focus on the relative contribution of basophils and DCs to Th2-mediated allergic responses.

## Introduction

T helper 2 (Th2) immune responses, which develop in response to allergens and parasites, are characterized by high levels of immunoglobulin E (IgE) and the presence of Th2 cells (1). Basophils are intimately involved in Th2 immune responses, and upon activation of the high-affinity receptor for IgE (FcεRI) or other surface receptors, they release multiple effector molecules, including proteases, vasodilating substances, such as histamine, cytokines, pro-inflammatory chemokines, and lipid mediators (2, 3). However, the mechanisms that initiate Th2 responses are not fully understood. Previous reports have shown that dendritic cells (DCs), the most efficient antigen-presenting cells (APCs) in the immune system, play a crucial role (4). However, recent experiments in newly developed basophil-deficient mouse models have highlighted the importance of basophils as well. For example, Th2 skewing is considered to be mainly induced by DCs, but recent studies in basophil-depletion mouse models indicate that basophils also play a pivotal role in this process (5–7). Here, we review the roles of basophils in cutaneous immune reactions and Th2-mediated allergic responses associated with cutaneous allergic diseases, focusing on the possibility that basophils and DCs function cooperatively in inducing Th2-mediated allergic responses.

## Cutaneous Allergic Diseases Associated with Basophil Infiltration

Basophils have been detected in the vicinity of eosinophils in several human cutaneous allergic diseases, and infiltration of basophils has been reported in several skin diseases, including atopic dermatitis (AD), prurigo, and urticaria (8). It is noteworthy that skin lesions of bullous pemphigoid, classical eosinophilic pustular folliculitis (Ofuji’s disease), and Henoch–Schönlein purpura also frequently exhibit tissue basophilia (8–11) (Table 1). We recently demonstrated the presence of both basophils and eosinophils in inflamed skin of patients with irritant contact dermatitis (ICD) (12). Further, we showed that basophils rapidly infiltrate into the inflamed skin, and subsequently induce infiltration of eosinophils with a croton oil-induced model of ICD. But it is still unclear exactly how basophils infiltrate into the lesional skin. There are several candidate basophil attractants, such as α(1,3)-fucosyltransferases IV and VII, for the initial recruitment of basophils in chronic allergic inflammation (CAI) (13). Subsequently, basophils attract eosinophils directly or indirectly via eotaxin-mediated interaction with mesenchymal fibroblasts (12).

**Table 1:** **Dermatological diseases accompanied with basophil infiltration**.

Atopic dermatitis (8)
Irritant contact dermatitis (12)
Prurigo (8)
Urticaria (8)
*Pemphigus vulgaris* (8)
Bullous vulgaris (8)
Drug eruption (8)
Henoch–Schönlein purpura (8)
Insect bite (tick bite) (11)
Scabies (8)
Dermatomyositis (8)
Eosinophilic pustular folliculitis (10)
Leprosy (LL type) (9)

## IgE-Mediated Chronic Allergic Inflammation and Chronic Idiopathic Urticaria

Immunoglobulin E-mediated chronic allergic inflammation (IgE-CAI) is a novel type of chronic inflammation of the skin that follows the immediate-type and late-phase responses in mice (14). It has been reported that IgE-CAI is independent of mast cells and T cells, but is dependent on basophils expressing FceRIa and CD49b phenotypic markers (14). Interestingly, although the number of basophils infiltrating the lesional skin is very low, their depletion led to a marked reduction in inflammation, concomitantly with decreased numbers of eosinophils and neutrophils and attenuation of the increased ear thickness (14). Recent studies have shown that inflammatory monocytes recruited to IgE-CAI lesions acquire an anti-inflammatory phenotype via basophil-derived IL-4 (15). Collectively, these results suggest a specific and non-redundant role for basophils in the initiation and maintenance of chronic IgE-mediated inflammatory responses in mice (16).

It is important to note that there are some functional differences between human basophils and mouse basophils. In mice, activated basophils produce platelet-activating factors and contribute to the development of anaphylaxis in response to penicillin-IgG antibody complexes. On the other hand, human basophils do not respond to IgG immune complexes (17, 18). Nevertheless, some findings in mouse basophils appear to shed light on the pathogenesis of human cutaneous diseases. Antibodies to FcεRIα were found in 40% of patients with chronic idiopathic urticaria (CIU). Some CIU patients exhibited urticaria in response to anti-FcεRIα IgG and/or IgE antibodies, which may stimulate mast cells or basophils (19). In addition, an activation marker on basophils, CD203c, was upregulated upon incubation of donor basophils with sera from patients with CIU (20). Furthermore, infiltration of basophils is increased in urticarial lesions of CIU (8, 21). Consistently with this finding, basopenia in CIU appears to be due to the migration of basophils from the peripheral blood to urticarial lesions (1, 8, 21). Therefore, the phenomena seen in the mouse IgE-CAI model might explain the pathogenesis of human CIU.

## Dendritic Cell-Specific and Basophil-Specific Depletion Models

Although the CD11c-based system is the most common depletion model of DCs, it has the disadvantage of imperfectly separating conventional DCs (cDCs) and macrophages (22, 23). On the other hand, since there are no natural mouse mutants with basophil deficiencies, antibodies that recognize either FcεRI (clone MAR-1) or the orphan-activating receptor CD200 receptor 3 (CD200R3) (clone Ba103) have been used to investigate the role of basophils. However, these antibody clones not only deplete basophils but also stimulate mast cells (24, 25). In addition, Ba103 activates myeloid cells and NK cells (26), and MAR-1 depletes a subset of FcεRI-positive DCs (27). Table 2 summarizes currently available mouse strains with constitutive or inducible depletion of basophils. Three groups have developed basophil-depletion models through regulation of *Mcpt8*, a basophil-specific gene in the conserved chymase locus (24, 28–30). In these mice, basophils were depleted in peripheral blood without side effects. A different basophil-depletion model utilizing the P1-Runx gene was reported by Mukai et al. (31) These mice show depletion of basophils, but not eosinophils, neutrophils, or mast cells. Sawaguchi et al. developed Bas-TRECK Tg mice, using a diphtheria-toxin receptor (DTR) transgene under the control of the DNase I-hypersensitive site 4 (HS4) region of IL-4 (32).

**Table 2:** **Mouse models of basophil depletion**.

Model system	Experimental strategy	Method of depletion	Depletion efficiency	Reference
Basoph8	Knock-in of IRES–YFP–Cre cassette before the Mcpt8 start codon	Cross to R-DTA mice	>90%	(28)
Mcpt8-Cre	BAC transgene (Cre inserted after the Mcpt8 start codon)	Constitutive depletion	>90%	(24)
Mcpt8^DTR^	Knock-in of IRES–DTR–EGFP cassette in 3′-UTR of Mcpt8	DT injection	>90%	(29)
P1-Runx1	Knockout	P1-Runx1 seems to be essential for the basophil lineage	>90%	(31)
Bas-TRECK	DTR transgene (under control of HS4 region of IL-4)	DT injection	>90%	(32)

*BAC, bacterial artificial chromosome; DTR, diphtheria toxin receptor; IL-4, interleukin-4; IRES, internal ribosome entry site; Mcpt, mast cell protease; NA, not applicable; NR, not reported; R-DTA, ROSA-diphtheria toxin-α; UTR, untranslated region; YFP, yellow fluorescent protein*.

## Basophils are Associated with Th2 Skewing in Response to Haptens

Mature DCs are generally considered to be required for naïve T cells to proliferate and acquire Th2 effector functions in response to antigen encounters (33). Recently, however, the function of DCs in Th2 induction has been questioned because basophils also appear to play a pivotal role in this process (5–7). Basophils migrate into draining lymph nodes (LNs) from the site of papain injection or helminth infection and act as APCs by taking up and processing antigens (5–7). In addition, basophils are capable of expressing MHC class II and costimulatory molecules, such as CD40, CD80, and CD86. They also secrete several cytokines critical for Th2 development, including IL-4 and thymic stromal lymphopoietin (TSLP). Thus, under certain conditions, basophils alone, without DCs, can cause Th2 induction from naïve T cells. However, the role of basophils in Th2 skewing has again been questioned since several of the above experiments used bone marrow-derived basophils (BMBaso) containing FcεRI-expressing inflammatory DC (27).

**Figure 1:**
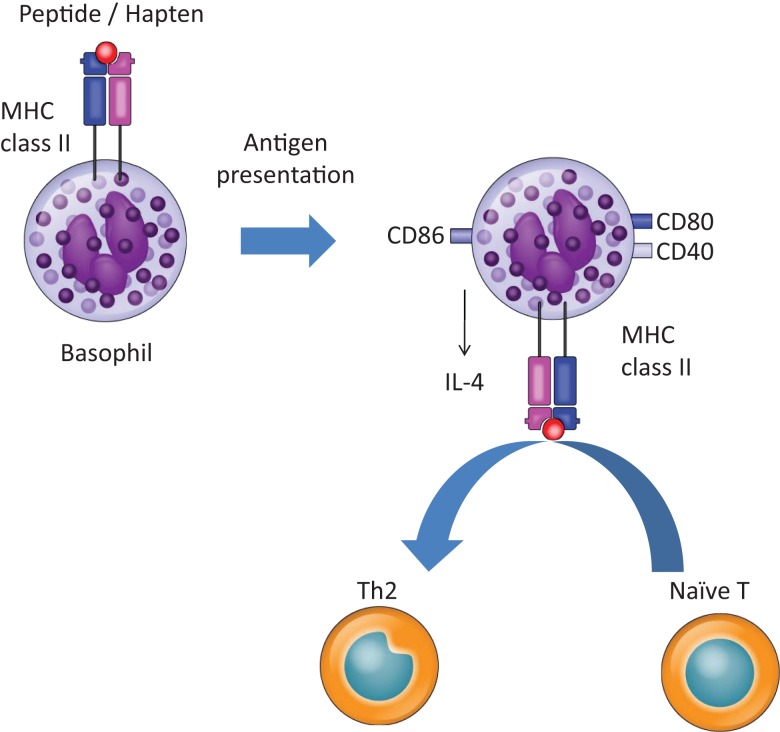
**Basophils promote Th2 skewing in response to haptens and peptide antigens**. Basophils promote Th2 skewing upon peptide and hapten exposure by expressing MHC class II, CD40, CD80, CD86, and IL-4.

**Figure 2:**
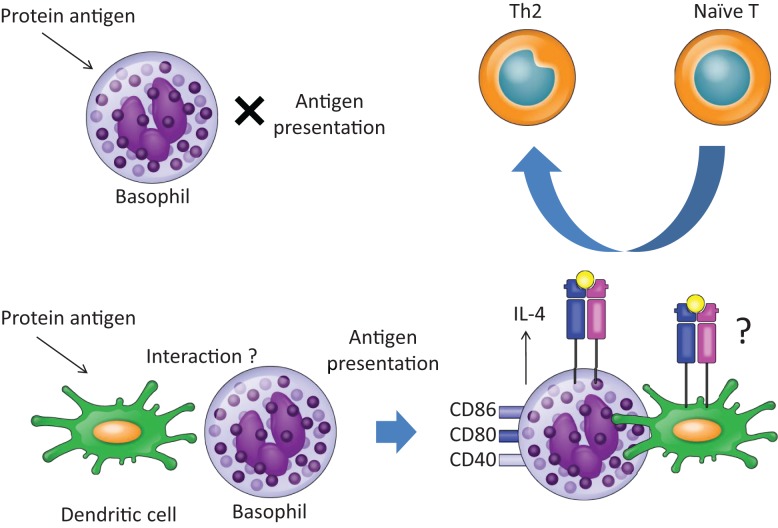
**Basophils promote Th2 skewing in response to protein antigens in the presence of dendritic cells**. Upon exposure to OVA protein, basophils do not work as APCs, since basophils cannot take up or process OVA protein. In the presence of dendritic cells, however, basophils promote Th2 skewing. The precise mechanisms of interaction between basophils and dendritic cells remain unclear.

Recently, we demonstrated that basophils play a role in Th2 skewing in response to haptens and peptide antigens, but not protein antigens, in a basophil-deficient mouse model, Bas TRECK Tg (34). In addition, we showed that basophils were capable of Th2 skewing by using CD11c-depleted BMBaso in order to avoid contamination with inflammatory DCs. Basophils express MHC class II, CD40, CD80, CD86, and IL-4 in the hapten-induced cutaneous Th2 model. However, using the DQ-OVA system, we confirmed that basophils did not efficiently take up or process protein antigens (34). A different experimental system using OVA coupled to fluorescein isothiocyanate showed that basophils could take up protein antigens (6), but our results showed that hapten antigens and peptides might bind directly to MHC class II on basophils, and they could be acquired and presented by basophils. On the other hand, basophils hardly digest protein and so cannot efficiently present protein antigens (Figures 1 and 2). Although the OVA-peptide system is totally artificial, complex inflammatory environments, such as post-*Schistosoma mansoni* or *Trichuris muris* infection, probably contain small soluble antigens as well as larger proteins. In addition, cutaneous immunization with papain protease allergen promotes MHC class II expression on basophils in LNs, probably after the generation of peptide antigens from the protein *in vivo* (6). A recent report showed that basophils are capable of inducing Th2 upon exposure to OVA proteins complexed with specific IgE (7). They pulsed basophils with various doses of DNP-OVA in the presence of monoclonal antibody to DNP (IgE anti-DNP) and showed that Th2 skewing by basophils was enhanced with the effect of IgE anti-DNP, especially when basophils were pulsed with low concentrations of DNP-OVA (7). House dust mites, which possess cysteine protease activity, are incapable of inducing Th2 when presented by basophils, even though cysteine protease may play a role in processing protein antigen into peptides *in vivo* (27), because the expressions of HLA-DM and of the invariant chain on basophils, those were sorted from the LNs 3 days after house dust mites administration, were very low (27).

Although several studies show that murine basophils can serve as APCs, the situation is less clear for human basophils. Human basophils express MHC class II (35, 36), but it was not able to induce antigen-specific T cell activation or proliferation in response to house dust mite allergen exposure (36). Another group reported that HLA-DR in human basophils is upregulated by IL-3 and IFN-γ, but the basophils cannot work as APCs for pollen allergen (37). It has been confirmed that human basophils lack some features of APCs (38, 39). Additional studies are needed to determine whether human basophils can act as APCs under various pathophysiological conditions.

## Interaction between DCs and Basophils for Th2 Skewing

It has been reported that basophils contribute to the strength of the Th2 response in the lungs, but they cannot present antigens or express chaperones involved in antigen presentation (27). Therefore, it was suggested that DCs are necessary and sufficient for inducing Th2 immunity to house dust mites in the lungs, and basophils are not required. In accordance with this idea, Th2 responses were severely impaired after *Schistosoma mansoni* egg injection and during active *Schistosoma mansoni* infection by depletion of CD11c^+^ cells, but not by depletion of basophils with anti-FcεRIα antibody (4). These findings suggest that some DC subsets induce Th2 skewing upon exposure to protein antigens.

Recently, two different groups have shown that Th2 skewing in response to *Nippostrongylus brasiliensis* infection depends on dermal CD301b^+^ DCs (40, 41). Depletion of CD301b^+^ DCs prior to infection reduces the number of IL-4-producing CD4^+^ T cells (40, 41). CD301b^+^ DCs also express programed death ligand-2 (PDL2), and a subset of PDL2^+^CD301b^+^ DCs that express the transcription factor interferon regulatory factor 4 (IRF4) was shown to be required for Th2 induction *in vivo* (41). In accordance with these findings, CD11c^+^MHC class II^+^ dermal DCs expressing PDL2, and CD301b were identified as a Th2-inducing DC subset in *Nippostrongylus brasiliensis* infection (42). However, CD301b^+^ DCs alone are incapable of inducing a Th2 response *in vitro* (41) or *in vivo* (40).

We have shown that basophils are capable of inducing Th2 skewing upon exposure to protein antigens in the presence of DCs (34). Because basophils are not able to take up or process protein antigens efficiently, DCs may prepare peptides from protein antigens for antigen presentation by basophils or may promote IL-4 production from basophils to skew Th2. In line with this, we had previously demonstrated that Langerhans cells, an epidermal DC subset, mediate epicutaneous sensitization with OVA protein antigen to induce Th2-type immune responses (43). Further studies are needed to show direct evidence whether DCs prepare peptides from protein antigens for the Th2 induction by basophils. In addition, Th2 reaction in response to schistosome infection or protein antigens was reduced in a CD11c-depletion model (4, 27). Therefore, DCs seem to be necessary for inducing Th2 reaction upon exposure to protein antigen both *in vivo* and *in vitro*.

Furthermore, basophils were found in the vicinity of T-cells in the T-cell zone of draining LNs by epicutaneous sensitization with haptens (34). Optimal localization of DCs within LNs may play a crucial role in Th2 skewing in each condition. CXCR5-expressing CD11c^+^ DCs migrate to the LNs and localize adjacent to B cell follicles in *Heligmosomoides polygyrus* infection, whereas depletion of CXCR5 or B cell-derived lymphotoxin alters the localization of DCs and impairs the development of Th2 cells (44). Therefore, although the location of DCs on draining LNs for Th2 induction is still controversial, it is possible that basophils, T cells, and DCs promote Th2 induction in a coordinated way. Similarly, reactive oxygen species (ROS) were generated in dermal DCs and in LN DCs upon subcutaneous exposure to papain plus antigen. ROS promoted Th2 response via formation of oxidized lipids that triggered TSLP production by epithelial cells. In addition, ROS enhanced Th2 induction by inducing release of CCL7 from DCs, leading to the recruitment of basophils to the draining LNs (45). Another group showed that IL-3 plays a role in basophil recruitment to draining LNs using helminth infection model with mice deficient in IL-3 or IL-3Rβ (46). However, they found that helminth-induced Th2 response was not diminished in an MAR-1 antibody-induced basophil-depletion model. Further studies are needed to determine whether DCs present peptides to basophils directly or whether plasma membrane fragments are transferred from APCs to lymphocytes by trogocytosis.

## Conclusion

Studies in basophil-deficient mouse models over the last decade have greatly improved our understanding of the mechanisms of development of Th2 immune reactions. Nevertheless, some key questions remain unanswered, including how DCs cooperate with basophils during Th2 skewing, especially in response to protein antigen exposure. In addition, the precise role of basophils in Th2 skewing, especially their function as APCs, remains controversial (27, 46). One possibility is that basophils may work as early IL-4-producing cells for the induction of Th2. An issue in some previous studies has been the imperfect separation of cDC in CD11c-based systems, and one possible approach to overcome this would be to use *CD11c*-DTR and *Zbtb46*-DTR, a marker specifically expressed by cDCs in lymphoid and non-lymphoid tissues but not by other myeloid or lymphoid cell types (47). Newly developed DC-deficient and basophil-deficient models are expected to provide further information on the mechanisms involved in Th2 skewing. Such studies may provide a basis for novel therapeutic approaches to controlling allergic diseases.

## Conflict of Interest Statement

The authors declare that the research was conducted in the absence of any commercial or financial relationships that could be construed as a potential conflict of interest.
